# Combining QTL mapping and gene co-expression network analysis for prediction of candidate genes and molecular network related to yield in wheat

**DOI:** 10.1186/s12870-022-03677-8

**Published:** 2022-06-13

**Authors:** Jun Wei, Yu Fang, Hao Jiang, Xing-ting Wu, Jing-hong Zuo, Xian-chun Xia, Jin-quan Li, Benjamin Stich, Hong Cao, Yong-xiu Liu

**Affiliations:** 1 Key Laboratory of Plant Molecular Physiology, Institute of Botany, Chinese Academy of Sciences, Beijing, 100093 China; 2 University of Chinese Academy of Sciences, Beijing, 100049 China; 3 National Wheat Improvement Center, Institute of Crop Sciences, Chinese Academy of Agricultural Sciences, Beijing, China; 4 Strube Research GmbH & Co., KG 38387 S ¨ollingen, Germany; 5 Institute of Quantitative Genetics and Genomics of Plants, Heinrich Heine University, D ¨usseldorf, Germany

**Keywords:** Grain size, Pre-harvest sprouting, Seed dormancy, Seed vigor, Spike length, WGCNA, Wheat

## Abstract

**Background:**

Wheat (*Triticum aestivum* L.) is an important cereal crop. Increasing grain yield for wheat is always a priority. Due to the complex genome of hexaploid wheat with 21 chromosomes, it is difficult to identify underlying genes by traditional genetic approach. The combination of genetics and omics analysis has displayed the powerful capability to identify candidate genes for major quantitative trait loci (QTLs), but such studies have rarely been carried out in wheat. In this study, candidate genes related to yield were predicted by a combined use of linkage mapping and weighted gene co-expression network analysis (WGCNA) in a recombinant inbred line population.

**Results:**

QTL mapping was performed for plant height (PH), spike length (SL) and seed traits. A total of 68 QTLs were identified for them, among which, 12 QTLs were stably identified across different environments. Using RNA sequencing, we scanned the 99,168 genes expression patterns of the whole spike for the recombinant inbred line population. By the combined use of QTL mapping and WGCNA, 29, 47, 20, 26, 54, 46 and 22 candidate genes were predicted for PH, SL, kernel length (KL), kernel width, thousand kernel weight, seed dormancy, and seed vigor, respectively. Candidate genes for different traits had distinct preferences. The known PH regulation genes *Rht-B* and *Rht-D*, and the known seed dormancy regulation genes *TaMFT* can be selected as candidate gene. Moreover, further experiment revealed that there was a SL regulatory QTL located in an interval of about 7 Mbp on chromosome 7A, named *TaSL1*, which also involved in the regulation of KL.

**Conclusions:**

A combination of QTL mapping and WGCNA was applied to predicted wheat candidate genes for PH, SL and seed traits. This strategy will facilitate the identification of candidate genes for related QTLs in wheat. In addition, the QTL *TaSL1* that had multi-effect regulation of KL and SL was identified, which can be used for wheat improvement. These results provided valuable molecular marker and gene information for fine mapping and cloning of the yield-related trait loci in the future.

**Supplementary Information:**

The online version contains supplementary material available at 10.1186/s12870-022-03677-8.

## Background

Bread wheat (*Triticum aestivum* L.) is one of the most important grain crops and widely cultivated worldwide [1]. In the face of global climate change, increasing wheat grain yield with limited land and water resources still meets great challenges [2]. Kernel weight is a major yield component in wheat determined by many components [3]. From the view of plant morphometric traits, increasing spike length (SL), kernel length (KL) and kernel width (KW) are the important approaches for breeding high-yielding wheat [4, 5]. Furthermore, wheat has a minimal seed-dormancy mechanism, which can cause pre-harvest sprouting (PHS) of seeds and significantly reduces grain yield and quality [6]. Another aspect is that long-term storage of seeds leads to low seed vigor with slow and non-uniform germination, resulting in poor yield in the following field season [7]. Thus, improvements of seed vigor and PHS resistance are also often targets in breeding [6, 7].

Wheat yield-related traits are typically controlled by multiple quantitative trait loci (QTLs). Previously, QTL mapping using various segregating population were conducted for wheat yield components. Cheng et al. [8] identified one major KL QTL on chromosome 2D that explained > 21.8% of phenotypic variances (PVE). Three kernel weight QTLs on chromosomes 2D, 4B and 5A were identified [9]. Furthermore, using a high-density genetic linkage map, five stable QTLs, including two for KL, one for KW and two for thousand kernel weight (TKW), were identified on chromosomes 2D, 5A, 5B and 6B [5]. Based on two biparental populations, Li et al. [4] identified six major spike compactness and length QTLs. These major QTLs co-located on chromosomes 5A and 6A, and explained 7.13–33.6% of PVE [4]. In previous study in wheat, we detected seed dormancy and vigor QTLs, of which four major QTLs for seed dormancy were found on chromosomes 3A, 3D, 6A and 7B [10], and two major QTLs for seed vigor were found on chromosomes 2D and 4A [11]. Due to the complex genome of wheat and the limited numbers of available markers, these QTLs were located in relatively large intervals of genetic map. However, constructing secondary populations and developing new molecular markers to fine-map the underlying gene are time-consuming.

QTL mapping has had success in identifying genes underlying plant quantitative trait. However, it requires genetic markers that specifically differentiate parental forms, controlled breeding and maintenance of large numbers of progeny [12]. Compared with QTL mapping, genome-wide association analysis (GWAS) that based on linkage disequilibrium, can investigate greater number of alleles and broader genetic variations in an association study [13]. In rice (*Oryza sativa*), *OsSPL13*, a grain yield controlling gene, had been identified through GWAS [14]. *bHLH16*, sharing a conserved function in regulating flag leaf angle, was identified by GWAS with 529 *O. sativa* accessions [15]. In addition to rice, for many major crops, such as maize (*Zea mays*) and soybean (*Glycine max*), agronomic trait had been study through GWAS [13, 16–19]. QTL mapping and GWAS are powerful tools to locate causal loci on the genome, but it is not enough to identify candidate genes on its own for complex traits. Thus, additional work is necessary. Causal genes of capsaicinoids that are unique compounds produced only in peppers (*Capsicum annuum*) were revealed by QTL mapping combined with GWAS [20]. Nitrogen is an essential element for plants, and genes related to nitrogen stress tolerance had been identified by combining QTL mapping and transcriptome profiling in sorghum (*Sorghum bicolor*) [21]. The marriage of transcriptomic approaches with genetic design has been proven a powerful tool in understanding of complex traits [22]. But such studies have rarely been carried out in wheat.

In this study, we had combined QTL mapping and weighted gene co-expression network analysis (WGCNA) to predict candidate genes related to yield in wheat. WGCNA is a systems biology method for describing the correlation patterns among transcription levels of genes across many samples [23]. It enables characterization of modules of co-expressed genes that may share biological function. Such networks provide an initial way to explore functional associations from gene expression profiling and can be applied to various aspects of plant biology [23, 24]. Wang et al. [25] analyzed the transcriptomes of developing spikes for 90 wheat lines and identified several genes related to spike complexity by WGCNA. *SmWRKY44*, an anthocyanin biosynthesis regulatory gene had been identified by WGCNA [26]. In tomato (*Solanum lycopersicum*), *SlHSP70–1* was connected with the *SlIAA9* and *SlDELLA* node in co-expression network, and overexpressing *SlHSP70–1* resulted in internode elongation [27]. In addition, WGCNA is less affected by homoeolog quantification, and is applicable to polyploids [28]. A combined integration of WGCNA with QTL mapping provided a multi-dimensional understanding of biological functional networks in this study. In this way, we predicted 29, 47, 20, 26, 54, 46 and 22 candidate genes for plant height (PH), SL, KL, KW, TKW, seed dormancy and seed vigor traits, respectively. Among them, three known genes, *Rht-B*, *Rht-D* and *TaMFT*, had been cloned as PH and seed dormancy regulator, respectively [29, 30]. Furthermore, a major QTL for multi-effect regulation of KL and SL, named *TaSL1*, was identified. It was located in the interval of about 7 Mbp on chromosome 7AS (7A short arm).

## Results

### QTL detection for plant height and yield-related traits

Using 241 F_10_ recombinant inbred lines (RILs) derived from Zhou8425B/Chinese Spring (CS) cross, we performed QTL mapping for PH, SL and seed related traits. Zhou8425B has a dwarf PH, large spike, big kernel and high TKW (Fig. S1), while CS has better PHS resistance and seed storability [10, 11]. Information about QTLs for seed dormancy and seed vigor were available from previous studies [10, 11]. The RILs were planted during the cropping season in 2016–2017, 2017–2018 and 2018–2019. ANOVA was conducted for phenotypes across the three environments. Significant differences among the 241 RILs were observed for the phenotypic values for all traits (Table S1). The frequency distributions of PH, SL, KL, KW and TKW for the RILs in each environment were continuous (Fig. S2), indicating that they are quantitative traits regulated by multiple genes. Based on data averaged across the three environments, PH ranged from 52.2 to 111.5 cm with an average of 85.1 cm. SL ranged between 6.71 and 13.82 cm with an average of 9.55 cm. TKW ranged from 21.8 to 44.1 g with an average of 33.5 g. KW and KL ranged from 2.37 to 3.44 mm and 5.34 to 6.86 mm, with averages of 2.95 and 6.00 mm, respectively. PH, SL, and KL showed high heritability, ranging from 0.74 to 0.79 (Table S1). Pearson’s correlation coefficients were calculated for all pairs of traits (Table S1, Fig. S3). The highest positive correlation was observed between TKW and KW (*r* > 0.85, *P* < 0.01). In addition, germination ratio (GR) and first count germination ratio (FCGR), which were used to reflect seed dormancy, had a high correlation (*r* > 0.73, *P* < 0.01). Weighted germination index (GI) and mean germination rate (MGR), which were used to reflect seed vigor, showed a high correlation (*r* > 0.65, *P* < 0.01, Fig. S3) as well. PH data from the three environments were highly correlated each other with *r* > 0.70 and *P* < 0.01, SL and KL also showed the similar correlations, indicating that they might be highly influenced by genetic factors (Table S1, Fig. S3).

**Fig. 1:**
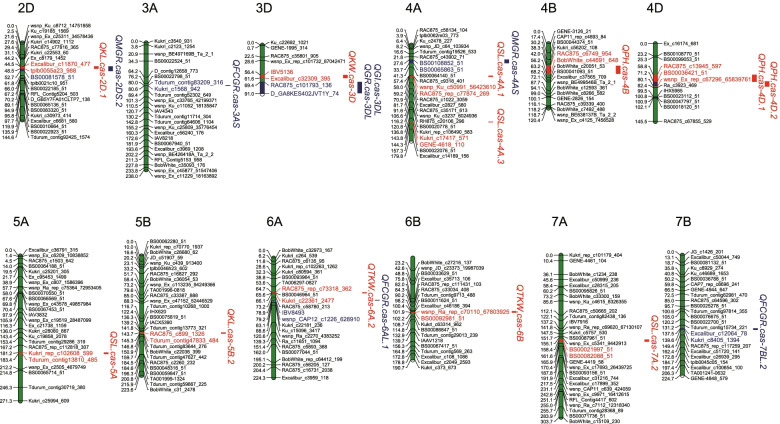
Genetic maps of chromosomes showing stable QTLs of wheat traits. The QTL identified in two of the three environments were designated as stable QTL. The QTLs for GI (weighted germination index) and MGR (mean germination rate), and the QTLs for GR (germination ratio) and FCGR (first count germination ratio) were quoted from our previous report, which were marked in blue. The other QTLs for plant height (PH), spike length (SL), kernel length (KL), kernel width (KW) and thousand kernel weight (TKW) were mapped in this experiment and marked in red

Using the SNP genotype and linkage map constructed previously [10], inclusive composite interval mapping (ICIM) identified 68 QTLs for PH, SL and seed traits across the three environments (Table S2). The QTLs identified in two of the three environments were designated in the following as stable QTL. Three QTLs for PH were stably identified across the three environments, and explained 13.7–23.2%, 8.6–18.5% and 7.2–7.5% of the PVE (Fig. 1, Table S2). Four stable QTLs for SL were identified on chromosomes 4A, 5A and 7A, and the *QSL.cas-4A.3* was detected in all environments and explained 9.3–11.6% of the PVE. Two stable QTLs for KL were detected on chromosomes 2D and 5B, and they explained 7.5–8.2% and 6.1–7.1% of the PVE, respectively. Fifteen QTLs for KW were identified, but only one stable QTL *QKW.cas-3D* was identified across the three environments, and explained 3.4–3.9% of the PVE. Furthermore, seven QTLs for TKW were detected on chromosomes 6A, 6B and 7B. Of these, the *QTKW.cas-6B* was detected in all of the three environments, explaining 6.9–7.8% of the PVE (Fig. 1, Table S2).

### Gene co-expression network analysis identified gene modules related to yield traits

The RNA sequencing (RNA-Seq) reads for the RILs were mapped to the CS genome (TGACv1, release-36). On average, 44.1 million reads pairs per sample were perfectly mapped to the genome. Using a criterion of reads per kilobase million (RPKM) value ≥0.5 for gene expression, 67,016 of 99,168 identified annotated genes were expressed in at least one RIL, whereas 25,458 annotated wheat genes were expressed across all RILs. A dendrogram of samples based on the RPKM values indicated that there were not any obvious outliers and the RILs can be divided into two subgroups (Fig. S4).

**Fig. 2:**
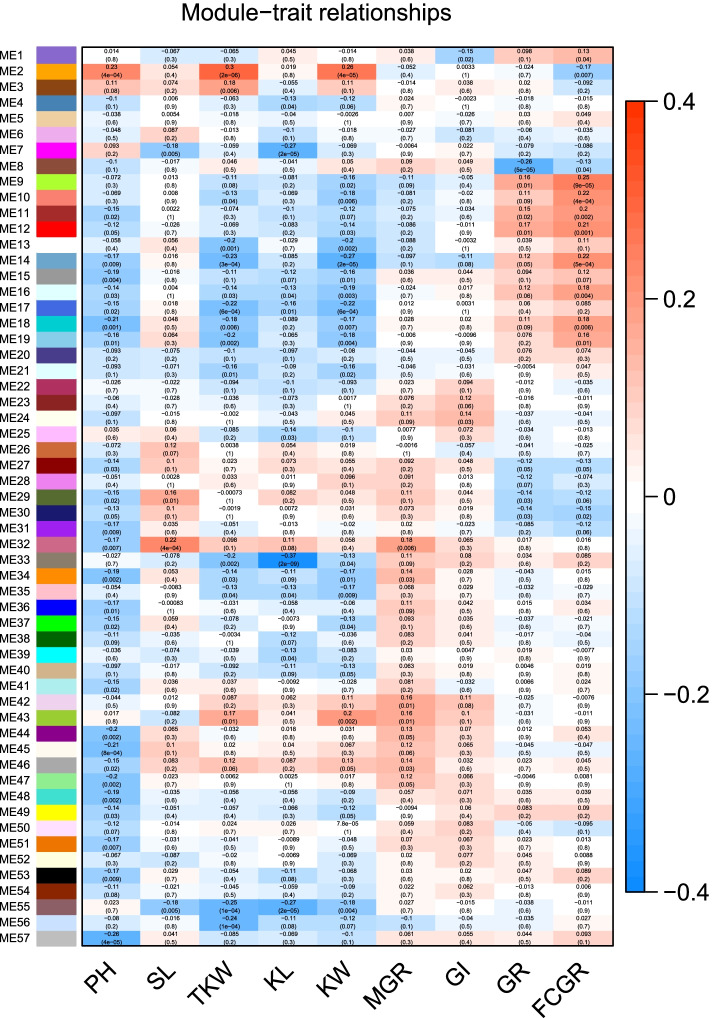
Module-trait relationship for 57 modules. Module-trait relationship of 67,016 expressed genes was analyzed by WGCNA package with parameter Soft-Threshold = 16 and minModuleSize = 50. Each row corresponds to a module eigengenes (MEs) that were defined as the first principal component of each gene module and the expression of MEs was considered as a representative of all genes in a given module, column corresponds to a trait. Each cell contains the corresponding correlation and *P*-value (in bracket). The table is color-coded by correlation according to the color legend. PH, plant height; SL, spike length; KL, kernel length; KW, kernel width; TKW, thousand kernel weight. MGR, mean germination rate; GI, weighted germination index; GR, germination ratio; FCGR, first count germination ratio

Next, a co-expression network was constructed using the WGCNA package for R [23] with the 67,016 expressed genes, followed by decomposition of the network into 57 subnetwork modules (Figs. 2 and S4). Each module contains a set of genes showing significant expression correlations with each other. The largest module ME48 contained 2533 genes, whereas the smallest module ME55 contained only 60 genes, and 40,554 ungrouped genes were assigned to module ME57 (Table S3). An eigen-value was calculated to represent the overall expression trend in each RILs for each module. For each module, the correlations between the eigen-value and the phenotypic values of all traits were computed across the 241 RILs. Interestingly, module ME2 exhibited a significantly positive correlation with PH, KW and TKW (*P* < 4.0e-4, Fig. 2). Therefore, Module ME2 may harbor major regulators influencing biomass accumulation in the entire period of wheat production. Besides, module ME32 was positively correlated with SL and KL, while module ME7 and ME33 was negatively correlated (Fig. 2). Therefore, module ME7 and ME32 may harbor antagonistic regulators influencing cell division or expansion in wheat. Module ME33 revealed the most significant negative correlation with KL (*r* = − 0.37, *P* = 2.0e-9). For seed vigor, module ME24, ME32, ME42 and ME43 were positively correlated with GI and MGR. For seed dormancy, module ME8 and ME30 were significantly negatively correlated with GR and FCGR, while for module ME9, ME12 and ME14, positive correlations were observed (Fig. 2). Moreover, lots of ungrouped genes in the module ME57, whose expression patterns were highly correlated with the traits, may act independently as regulators (Fig. 2, Table S3).

### Combining QTLs and co-expression network analysis to predict candidate genes for yield traits

**Fig. 3:**
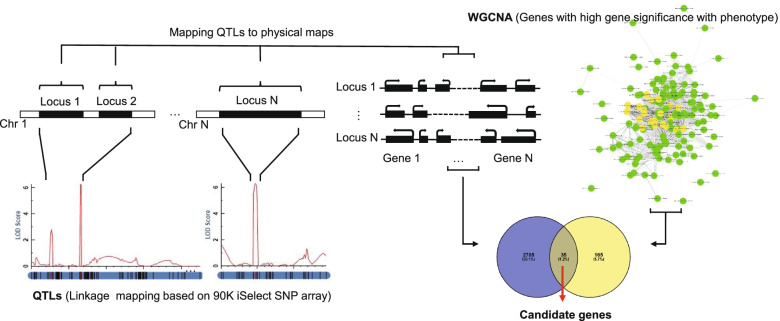
Schematic diagram of candidate gene prediction. Firstly, selecting the major effect QTLs that were detected in at least two of the three environments. Secondly, based on the sequence of markers, mapping QTLs to physical map, and all genes in the QTL interval were selected. Thirdly, the genes from highly associated modules or with high GS value (GS was defined as mediated *P*-value of each gene in the linear regression between gene expression and the traits, including the highest and lowest 200 genes) were selected based on WGCNA. Finally, the joint genes identified through the second and third procedure were considered as candidate genes

We identified candidate genes based on the strategy combining the QTL mapping and WGCNA. We first mapped the stable QTLs to physical map based on the sequence of markers and the genes with high phenotypic correlation in corresponding QTL were designated as candidate genes (Fig. 3, Table S4). In this way, 29, 47, 20, 26, 54, 46 and 22 candidate genes were predicted for PH, SL, KL, KW, TKW, seed dormancy and seed vigor, respectively (Table S4).

**Fig. 4:**
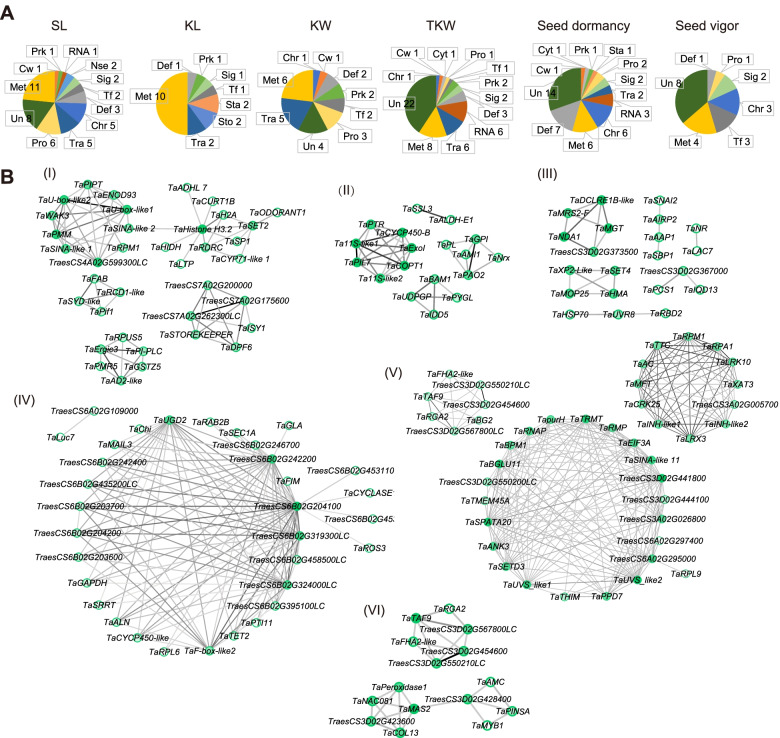
Candidate gene function classification and network prediction. **A** The functional classification of candidate genes was based on sequence BLAST with the Non-Redundant Protein Sequence Database (Nr), KEGG orthology (KO) and the Gene ontology (GO) database. Abbreviations for functional classes are as follows: Cyt, cell cytokinesis and cytoskeleton; Chr, chromatin binding; RNA, RNA binding; Tf, transcription factor; Sig, signaling; Def, defense or response to stresses; Cw, cell wall; Sto, storage proteins; Met, metabolism (enzymes); Nse, nuclease-like; Tra, membrane protein or transport, including protein, RNA or other material transport; Un, hypotheticals/unknowns/unknown function; Pro, protease or ubiquitin proteasome; Prk, Protein kinase-like; Sta, starch synthesis. **B** Predict the internal network of candidate genes for six different phenotypic traits. Darker green of node showed higher degree of network. Darker black of edge showed higher weight. (I) candidate genes for SL; (II) candidate genes for KL; (III) candidate genes for KW; (IV) candidate genes for TKW; (V) candidate genes for Seed dormancy; (VI) candidate genes for seed vigor

The functional classification of these candidate genes was based on sequence BLAST with the Non-Redundant Protein Sequence Database (Nr), KEGG orthology (KO) and the Gene ontology (GO) database. These genes were functionally divided into 15 categories (Fig. 4A). Candidate genes functions of different traits had different preferences. The unknown (Un) and metabolism (enzymes, Met) related candidate genes were involved for all traits (Fig. 4A, Table S4). The PH candidate genes belonged apart from Un and Met to the following categories: the most were cell cytokinesis and cytoskeleton (Cyt), and membrane protein or transport (Tra). Interestingly, the known PH genes *Rht-B* (*TraesCS4B02G043100*) and *Rht-D* (*TraesCS4D02G040400*) were detected by our approach (Table S4), which suggested that our strategy was effective. Similarly, among the candidate genes for SL, protease or ubiquitin proteasome (Pro), Tra and chromosome binding (Chr) were abundant. The candidate genes of KL mainly belonged to the category Tra, storage proteins (Sto) and starch synthesis (Sta) apart from Met. Most of KW candidate genes belonged to Pro and Tra (Fig. 4A). For TKW candidate genes, in addition to Un and Met, genes related to Tra, RNA binding (RNA) and defense or response to stresses (Def) were abundant. For seed dormancy, candidate genes related to Def, Chr and RNA were dominant and a known seed dormancy gene *TaMFT* (*TraesCS3A02G006600*) was identified. Among the candidate genes for seed vigor, genes mainly belonged to transcription factor, Chr and Signaling (Fig. 4A, Table S4).

We predicted gene network for candidate genes using WGCNA package and Cytoscape [31] based on gene expression pattern (Fig. 4B). For SL, 36 of 47 candidate genes were assigned to four subnets (Fig. 4B I). *TaSINA-like*, *TaHistone H3.2* and *TaU-box-like* had a higher degree of network, which implied that they may have important SL regulatory effects. The candidate genes for KL, *TaCYCP450-B* and *TaPIL7*, had a higher degree of network and connected to seed storage protein genes *Ta11S-like1* and *Ta11S-like1*. In addition, *TaBAM1,* a serine/threonine-protein kinase gene, connected to *TaUDPGP* that is involved in energy metabolism regulation (Fig. 4B II). For KW, 21 of 26 candidate genes were assigned to six subnets (Fig. 4B III), which implied KW may be regulated by many pathways. A total of 54 candidate genes of TKW were detected, which is the most among all traits. This may be related to *QTKW.cas-6B* locating in a large interval (Table S4). Interestingly, most of TKW candidate genes were assigned to a network in which each gene had high degree of network and weight values (Fig. 4B IV). It suggested that these genes work closely together to regulate TKW. Seed dormancy candidate genes were divided into three subnets. *TaMFT*, a known seed dormancy regulatory gene, had higher degree of network. In addition, *TaEIF3A*, *TaTRMT* and *TaSINA-like 11* in another subnet had a higher degree of network (Fig. 4B V). For seed vigor, candidate genes were divided into three subnets and these genes were related to each other and may jointly regulate seed vigor (Fig. 4B VI).

**Fig. 5:**
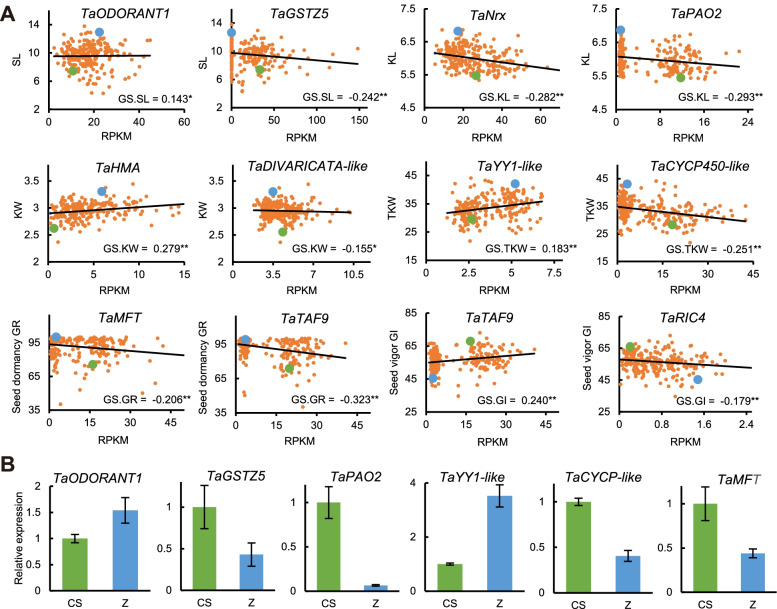
Expression analysis of candidate genes between parent Chinese Spring and Zhou8425B. **A** The relationship between gene expression level and traits variation. The gene expression level was reflected by RPKM value. The green dot indicates Chinese Spring and the blue dot indicates Zhou8425B. The gene significance (GS) was defined as mediated *P*-value of each gene in the linear regression between gene expression and the traits. *, Statistical test *P*-value is significant at the 0.05 level. **, Statistical test *P*-value is significant at the 0.01 level. SL, spike length (cm); KL, kernel length (mm); KW, kernel width (mm); TKW, thousand kernel weight (g); GR, germination ratio (%); GI, weighted germination index (%). **B** Using qRT-PCR to verify the expression levels of candidate genes between parent Chinese Spring and Zhou8425B. CS, Chinese Spring; Z, Zhou8425B. Total RNA was extracted from 21 DPA wheat spike. Gene expression was calculated using the 2^-ΔΔCt^ methods with *TaActin* as an internal control. Data are means (±SE) of three biological replicates, *n* = 3

We selected some candidate genes that had high gene significance (GS) which is defined as mediated *P*-value of each gene in the linear regression between gene expression and the trait. (Fig. 5A). These genes included transcription factors, such as *TaODORANT1*, *TaYY1-like* and *TaDIVARICATA-like*, and chromosomal binding genes et al. The expression pattern of *TaYY1-like* was positively correlated with TKW, while *TaCYCP450-like* was negatively correlated. *TaTAF9* was both a candidate gene for seed dormancy and seed vigor. The higher its expression level, the stronger seed dormancy and higher seed storage tolerance (Fig. 5A). To verify the RNA-Seq results, we selected some candidate genes for expression analysis between parent CS and Zhou8425 using quantitative RT-PCR (qRT-PCR) (Fig. 5B). The expression patterns of these genes were generally consistent with RPKM value from RNA-Seq (Fig. 5), which suggested that the RNA-Seq data were credible.

### Gene markers on chromosome 7A were tightly associated with SL and KL

**Fig. 6:**
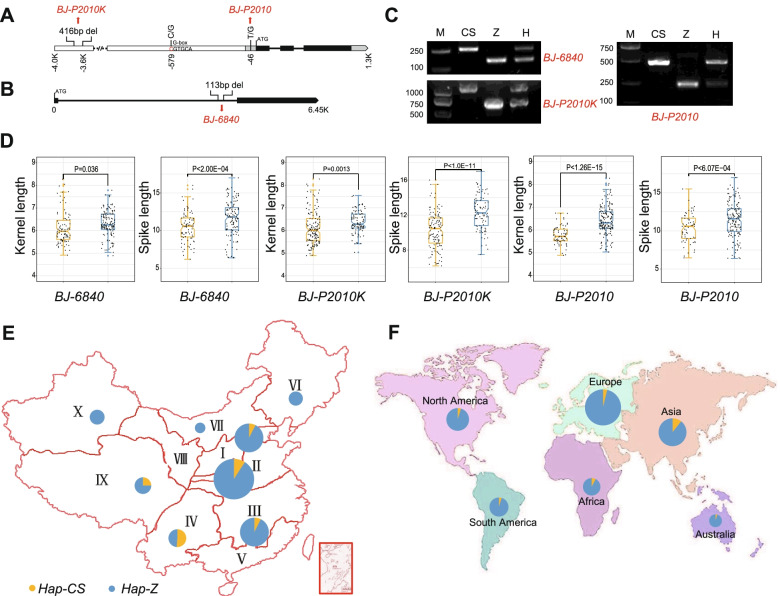
*TaSL1* regulated both spike and kernel length and the distribution of its preferred haplotype. **A** Gene marker *BJ-P2010K* and *BJ-P2010* was developed based on a 416 bp deletion and a SNP in the promoter of *TraesCS7A02G205100*, respectively. The solid black box represents exons and the solid black line represents introns; The gray box represents 5′-UTR and the gray arrow box represents 3′-UTR; The white box and dotted line represents promoter. **B** Gene marker *BJ-6840* was developed based on a 113 bp deletion in fist intron of *TraesCS7A02G200000*. **C** Agarose gel plots of gene markers. Upper left picture shows marker *BJ-6840*; Bottom left picture shows marker *BJ-P2010K*; Right picture shows the digestion of PCR product of primer set BJ-P2010-F/R with enzyme *Sma*I. The plots of different markers were from different gels. The completed gels are shown in Figure S6. M, molecular marker; CS, genotype of Chinese spring; Z, genotype of Zhou8425B, H, genotype of heterozygous. **D** Gene markers *BJ-P2010K*, *BJ-P2010* and *BJ-6840* were associated with both spike length and kernel length in 265 wheat landraces. **E** Distributions of two haplotypes of *TaSL1*(represented by *BJ-P2010*) in 429 cultivars population in different ecological regions of China. I, North winter wheat zone; II, Huanghuai winter wheat zone; III, The Yangtze river winter wheat zone; IV, Southwest winter wheat zone; V, South China winter wheat zone; VI, Northeast spring wheat zone; VII, North spring wheat zone; VIII, Northwest spring wheat region; IX, Qinghai-Tibet Spring-Winter Wheat zone; X, Xinjiang winter-spring wheat zone. **F** Distributions of two haplotypes of *TaSL1* in 334 varieties from the six worldwide wheat production regions (Excluding China). The relative size of the pie chart indicates the relative size of the sample

The gene and promoter sequences of the candidate genes for the QTL *QSL.cas-7A.2* were sequenced. Among them, two candidate genes, an unknown gene (*TraesCS7A02G200000*) and *TaODORANT1* (*TraesCS7A02G205100*), have abundant genetic variation between CS and Zhou8425B. Between both parental inbreds, 16 SNPs and one 416 bp deletion in promoter of *TaODORANT1* (Figs. 6A and S5), and one 113 bp deletion in the first intron of *TraesCS7A02G200000* were detected (Fig. 6B). We designed molecular markers based on these variations (Figs. 6C and S6). Using a population comprising 265 wheat landraces from around the world (Table S5), an association analysis was performed between these markers and the two yield components SL and KL. The result suggested that the markers *BJ-P2010* and *BJ-P2010K* for *TaODORANT1*, and *BJ-6840* for *TraesCS7A02G200000* were significantly associated with SL and KL (*P* < 0.01, Fig. 6D, Table S5). Gene markers were developed for seven additional indels on both sides of marker *BJ-6840* based on sequence difference between Zhou8425B and CS (Fig. S7, Table S6). These markers were also significantly associated with SL and KL in the above-mentioned panel of 265 landraces (Fig. S7, Table S5). Thus, we hypothesized there were significant SL and KL regulators in this region (159.4–167.2 Mbp) (Fig. S7), which were named *TaSL1*.

In order to investigate *TaSL1* application in wheat improvement, a total of 879 wheat accessions were genotyped with gene markers mentioned above. The 879 accessions comprise 429 cultivars from China, 185 cultivars from other zones and the 265 landraces mentioned above (Table S5). The results indicated that from landraces to cultivars, the ratio of the preferred haplotype of *TaSL1* (*Hap-Z*) was increased from 30.2 to 89.3% (*BJ-P2010* as representative, Table S5). Furthermore, the increase was observed in all ecological wheat zones, especially in the major production zones I, II and III (Fig. 6E). In addition, the frequency of the advantageous allele in varieties from the other six major wheat production continents around the world were 90.3, 86.5, 94.4, 95.9, 91.8 and 95.3%, respectively (Fig. 6F). The high ratios of preferred haplotypes in American, European and Australian wheat might be due to their longer breeding histories.

## Discussion

In wheat kernel development, the soft dough stage is generally about 21 days post anthesis (DPA). During this stage, the kernel size reaches its peak [32], and the transcript levels of genes encoding for storage proteins and defense proteins generally reach their maximum and tend to be maintained until the end of maturation [33]. Furthermore, many lipid peroxidation and metabolic enzymes closely related to seed vigor are most active during this stage [34, 35]. Cytokinin levels in the kernel are at their highest before milk stage and the level decreases gradually until it reaches the minimum at about 20 DPA, while abscisic acid (ABA) level is low in the initiation stage of kernel development, and reaches its highest level in the soft dough stage, and gradually decreases in the following stages [32, 36, 37]. The balance of ABA and GA plays an important role in seed dormancy. Studies have shown that when the dry matter content of grain reaches 45%, the germination difference between resistant and non-resistant PHS can be observed [38]. This implies that seed dormancy is basically formed at the soft dough stage. Thus, in the analysis of gene co-expression network for related yield traits in this study, the whole spike at 21 DPA were used for RNA-Seq.

In this study, we first performed QTL mapping for PH and yield-related traits. The QTLs were identified on chromosomes 2D, 3D, 4A, 5A, 5B, 6A, 6B and 7A (Fig. 1, Table S2). Among them, QTLs on chromosomes 2D, 4A, 5A, 6A were consistent with previous studies [4, 39–41]. However, the functional genes of these QTLs in wheat are still unknown. Based on a combined strategy of QTL mapping and WGCNA (Fig. 3), we predicted candidate genes for them. Three known genes, including two PH regulating genes (*Rht-B* and *Rht-D*) and one seed dormancy regulating gene (*TaMFT*), had been cloned previously [29, 30].

The wheat SL regulation genes are rarely reported. In rice, the known main panicle length regulatory genes are *DEP2* (*Dense and erect panicle 2*) [42] and *SP1* (*Short panicle1*) [43]. *SP1* encodes a peptide transporter [43]. *DEP2* encodes an unknown plant-specific protein and is highly expressed in young panicles [42]. In our study, multiple genes associated with the ubiquitin-proteasome pathway were selected, such as *TaU-box-lik*e and *TaSINA-like* (Fig. 4B, Table S4). They were all located on chromosome 4A and may be candidate genes of *QSL.cas-4A.1* and *QSL.cas-4A.3*. In addition, two genes located in the QTL *TaSL1*, an unknown gene and a MYB transcription factor gene *TaODORANT1*, were predicted for SL candidate genes (Fig. 4B, Table S4). A previous study showed that *TaODORANT1* participates in seed storage protein synthesis in wheat [44]. Our results showed that its promoter has several SNPs in Zhou8425B compared to CS (Figs. 6 and S5). Based on the finding, we further identified that QTL *TaSL1* was associated with both SL and KL (Figs. 6 and S7). The *TaSL1* had been widely selected in breeding (Fig. 6 E, F), but it still has important value for wheat improvement in some regions, such as Asia and Africa, especially for Southwest winter wheat zone and Qinghai-Tibet Spring-Winter Wheat zone in China (Fig. 6 E, F). Lots of cultivars in these areas, such as Zang1941, Yu23, Yu615, Mianmai1403, Mianmai185, Mianmai46, Mianyang16, Mianyang20, Mianyang26, etc., (Table S5) do not contain the preferred haplotypes of *TaSL1*, and they may still have the potential for improvement.

Previously, wheat kernel size or weight regulator were identified by homologous cloning or reverse genetics, such as *TaGW2*, *TaGS5* and *TaTGW6* [45]. These genes are mainly associated with three genetic pathways: G-protein signaling, phytohormone signaling and proteasomal degradation [45]. In our study, *TaPTR*, *TaCYCP450-like* and *TaAMI1* were selected as candidate genes (Fig. 4B, Table S4). They may regulate grain size or weight by participating in plant hormone signaling. *Arabidopsis* AtPTR has the capability of transporting plant hormones including auxin, ABA and GA [46]. AMI involved in auxin biosynthesis [47]. And most brassinosteroid biosynthetic enzymes belong to the cytochrome P450 family [48]. Besides, in our results, many genes involved in the proteasomal degradation were selected, such as *TaPCS1*, *TaAIRP2*, *TaCLPX* (Fig. 4B, Table S4).

Seed dormancy affects PHS resistance. The known genes involved in PHS regulation in cereals include *TaMKK3*, *TaMFT*, *TaSdr* and other regulators related to the signaling pathways [6]. Gene classification based on BLAST showed that seed dormancy candidate genes were mainly divided into two categories, one could be related to chromatin or DNA binding, such as *TaRPA1*, *TaTAF9* and *TaFHA2-like*, and the other could be related to RNA regulation, such as *TaEIF3A*, *TaRPS5* and *TaUTP21*, (Fig. 4, Table S4). These genes may regulate seed dormancy by sensing the external environment or endogenous signals. In addition, *TaMFT* was selected and had high degree of network among the candidate genes (Fig. 4B, Table S4). Two invertase inhibitor genes, *TaINH-like1* and *TaINH-like2*, were selected for seed dormancy candidate gene and connected with *TaMFT* in subnet (Fig. 4B). Invertase inhibitors classified as cell wall/apoplastic and vacuolar belonging to the pectin methylesterase family, play a major role in plant development and responses to environment, including seed germination [49, 50].

Lipid peroxidation is a major causes of seed vigor decline [33]. A series of causal genes had been identified in rice, such as *LOX2/LOX3*, *ALDH7* and *AKR*, etc. [33]. Among our predicted candidate genes, *TaPeroxidase1* and *TaPeroxidase2* that were related to peroxidation, and *TaMAS2* were detected (Fig. 4B, Table S4). *TaMAS2* is a momilactone A synthase-like gene which may function in α-Amylase and α-Glucosidase activity regulation [51]. In addition, six candidate genes, *TraesCS3D02G454600*, *TraesCS3D02G550210LC*, *TraesCS3D02G567800LC*, *TaFHA2-like*, *TaTAF9* and *TaRGA2* were selected for both seed vigor and seed dormancy (Fig. 4B, Table S4). FHA domain-containing proteins localize to the nucleus, where they participate in establishing or maintaining cell cycle checkpoints, DNA repair, or transcriptional regulation [52]. TAFs are components of the transcription factor IID complex that are essential for the regulation of RNA polymerase II-mediated transcription [53].

## Conclusions

In summary, a total of 68 QTLs were identified for PH, SL and seed traits, and 12 QTLs were stably identified across the three environments (Fig. 1). By a combined use of QTL mapping and WGCNA (Fig. 3), 29, 47, 20, 26, 54, 46 and 22 candidate genes were predicted for PH, SL, KL, KW, TKW, seed dormancy, and seed vigor, respectively. Candidate genes for different traits have distinct preferences. The known PH regulation genes *Rht-B* and *Rht-D*, and the known seed dormancy regulation genes *TaMFT* can be selected, which suggested that the integrated strategy was effective. Moreover, further experiment revealed that there was a SL regulatory QTL on chromosome 7A, named *TaSL1*. *TaSL1* was located in an interval of about 7 Mbp, which also regulated KL. It has important value for wheat improvement. These results provided valuable molecular marker and gene information for fine mapping and cloning of the yield-related trait loci in the future.

## Methods

### Wheat planting and phenotyping

A total of 241 F_10_ RILs derived from the cross of Zhou8425B/CS and 879 wheat accessions were used in this study (Table S5). The 879 wheat accessions consisted 429 cultivars from China, 185 cultivars from other zones and 265 landraces form around the world. Zhou8425B is an elite Chinese wheat line developed by the Zhoukou Academy of Agricultural Sciences in 1984, having a semi-dwarf PH, large spike, high TKW and multiple disease resistance [39]. CS has better PHS resistance and seed storability compared to Zhou8425B [10, 11]. For phenotyping, all 241 RILs (including parents, s285, CS; s377, Zhou8425B) were planted at the Experimental Station of the Institute of Botany, Chinese Academy of Sciences, Beijing during the cropping season in 2016–2017, 2017–2018 and 2018–2019, respectively. The 879 wheat accessions were planted in 2019–2020. All lines were planted in a randomized complete block design with two replications for each year. Planting row width was 1.5 m with 20 cm between rows. A total of 30 seeds were sown evenly in each row. The field traits were managed following the local normal practices.

The same phenotyping procedure were applied in each environment. PH and SL were measured in the field at 21 DPA with 12 randomly selected plants and spikes per line. The mean value was used as the final result. Yield-related traits, such as KL, KW and TKW, were measured in the laboratory following harvest by SC-G system (Hangzhou Wanshen Detection Technology Co., Ltd., Hangzhou, China, www.wseen.com). Seed dormancy (GR and FCGR) and seed vigor (GI and MGR) phenotype data were available from our previous work [10, 11].

### Genome-wide linkage mapping of QTL for PH and yield-related trait

The 241 RILs, including their parents, were genotyped with the 90 K iSelect SNP array from CapitalBio Corporation (http://www.capitalbio.com). SNP genotyping and linkage map come from Gao et al. [39]. QTL analysis was performed using ICIM with IciMapping 4.1 software [54]. Phenotypic values of all lines in each environment were used for QTL detection. The walking step chosen for all QTL was 1.0 cM, with *P* = 0.001 in stepwise regression. Mapping method was ICIM-ADD. A LOD threshold of 2.5 was chosen for declaration of putative QTL [55]. The PVE was estimated through stepwise regression [39].

### RNA-Seq and gene co-expression network analysis

In May 2017, during the soft dough stage of wheat (21 DPA) from the above described field experiment, the whole spike of 241 lines of RILs were collected and stored at − 80 °C for RNA extraction. Total RNA was extracted using a Plant Total RNA Purification Kit (GeneMark). Nanodrop 2000 and the Agilent 2100 bioanalyzer were used to characterize RNA quality. The purified RNAs were used to construct libraries and sequenced on Illumina HiSeqTM 4000 platform. Raw reads were examined using fastQC to inspect read qualities and the extent of adapter sequence contamination. Adapter sequences and reads with qualities of less than 20 were trimmed. Reads from a pair where only one read passed the filtering criteria were omitted. After pre-processing, all remaining reads were aligned to the CS genome (TGACv1, release-36) using TopHat [56], allowing a maximum of five mismatches. We calculated the number of uniquely mapped reads for each gene model in the CS genome by parsing the alignment output files from TopHat, and then normalized the resulting read counts by RPKM to measure the gene expression level. Low abundance genes with an expression cut off of RPKM < 0.5 in all line were removed from the set.

Scale-free co-expression network analysis was performed based on RPKM values of expressed genes using the WGCNA package (v 1.51) in R with parameter minModuleSize = 50 and Soft-Threshold = 16 (as determined by assessment of scale-free topology, Fig. S4). For network construction, we used a dynamic tree cutoff 0.20 to merge similar trees. To identify networks associated with trait variables, we calculated the eigen-value of each module, after which Spearman’s rank correlation was calculated between the eigen-value (overall expression trend of the genes in each module) and traits. Those with a weight value less than 0.02 were filtered out to construct candidate gene network. The Cytoscape software was used to draw the visual network [31].

### Candidate genes prediction for stable QTLs

The candidate genes for stable QTLs were predicted based on a combination of QTL mapping and WGCNA (Fig. 3). Firstly, we selected the major QTLs that were detected in at least two of the three environments. Secondly, based on the sequence of markers, QTLs were positioned to physical map, and all genes in corresponding QTL interval were selected. Thirdly, the genes from highly associated modules or with high GS value (Including the highest and lowest 200 genes for each trait) were selected based on WGCNA. Finally, the joint genes identified through the second and third procedure were considered as candidate genes.

The functional classification of candidate gene was based on sequence BLAST with the Nr, KO and GO database using EggNOG (http://eggnog5.embl.de/). For unannotated candidate genes, we used Batch CD-Search of NCBI (https://www.ncbi.nlm.nih.gov/) to search their conserved domains and define their function.

### Quantitative RT-PCR

RNA samples used for qRT-PCR were isolated from the whole spike at soft dough stage (21 DPA) of wheat that collected from three biological replicates. Gene-specific primers were designed using Primer Premier 5.0 software and are listed in Table S6. The first-strand cDNA was synthesized by using a FastQuant RT Kit (TIANGEN, Beijing) according to the manufacturer’s protocol. Amplification via PCR was done following the instructions of the KAPA SYBR® FAST qPCR Kit (Sigma, USA), performed following the fluorescent quantitative PCR amplification instrument (EppendorfMaster™ ep realplex, Germany). Gene expression was calculated using the 2^-ΔΔCt^ methods with *TaActin* as an internal control. Non-specific products were identified by melting curve analysis. Each sample included three technical replicates.

### Molecular marker development and association analysis

Sequence variation were detected for candidate genes between Zhou8425B and CS using sanger sequencing of PCR products. After sequencing, the sequences were aligned by DNAMAN (http://www.lynnon.com/). The marker *BJ-P2010K* and *BJ-P2010* were developed to discriminate the two haplotypes at *TaSL1* between CS and Zhou8425B. For *BJ-P2010K*, *Hap-Z* that is the haplotype of Zhou8425B has a deletion of 416 bp at position − 3.6 Kbp, Thus, marker *BJ-P2010K* was developed based on PCR product size. Genome specific primer set BJ-P2010K-F/R were used to amplify fragments in all lines. The fragments length of *Hap-CS* was 1142 bp, whereas *Hap-Z* was 728 bp (Figs. 6 A, C and S6). Similarly, *BJ-6840* marker was developed to discriminate the two haplotypes at its locus (Fig. 5 B, C). The CAPS marker *BJ-P2010* was developed based on the SNP (T/G) at position − 46 bp. The 460 bp specific fragment was amplified using primer set BJ-P2010-F/R, of which, *Hap-Z* could be cleaved by restriction endonuclease *Sma*I (222 bp and 238 bp, Fig. 5 A, C). In addition, seven additional indels on both sides of marker *BJ-6840* based on sequence difference between Zhou8425B and CS were developed as gene markers. The detailed information of each molecular marker and primer is shown in Table S6.

Young leaves of wheat were used for DNA extraction with CTAB method [57]. All ten markers at QTL *TaSL1* were used to performed association analysis using the 265 wheat landraces from around the world (Table S5). The phenotypic mean values were used as phenotypic data. Two-tailed *T*-test was used for exam whether the two haplotypes (*Hap-CS* and *Hap-Z*, and *Hap-CS* is the haplotype of CS) were different from one another for traits. The marker *BJ-6840* and *BJ-P2010* were used to investigate *TaSL1* application in wheat improvement (A total of 429 cultivars were used for China and 334 varieties were used for other zones).

### Statistical analysis

Numerical values were presented as means ± SE. Statistical analysis was performed using one-way ANOVA. The phenotypic correlation was analyzed using the rcorr tool in the Hmisc package of R with type = “pearson”. Two-sample *F*-test for variances was used to determine if the variances of the two haplotypes are equal, and two-tailed t-test was used for exam whether the two haplotypes are different from one another for traits. The broad hereditary capacity (*h*^*2*^) for each trait was estimated according to the method described by Smith et al. [58]. The ggplot2 (https://ggplot2.tidyverse.org/index.html) package of R was used for drawing.

## Supplementary Information

**Additional file 1: Figure S1.** Chinese wheat Zhou8425B and Chinese Spring. **Figure S2.** Frequency distributions of PH, SL, KL, KW and TKW for the RILs in three environments. **Figure S3.** Correlation analysis among the nine phenotypes. **Figure S4.** Weighted gene co-expression network analysis for expressed genes of the RILs. **Figure S5.** Promoter sequence alignment of *TaODORANT1* between wheat Chinese spring and Zhou8425B. **Figure S6.** The full-length agarose gel plots of gene markers. **Figure S7.** The gene markers on QTL *TaSL1* were significantly associated with spike length and kernel length.

**Additional file 2: Table S1.** Analysis of variance and Pearson correlation for phenotypes.

**Additional file 3: Table S2.** QTLs for plant height and yield traits.

**Additional file 4: Table S3.** WGCNA result for 67,016 genes.

**Additional file 5: Table S4.** Predicted candidate genes and their information for each trait.

**Additional file 6: Table S5.** Genotyping of each gene marker in wheat varieties. The visualization results are shown in Fig. 6 and Figure S7.

**Additional file 7: Table S6.** The primer sequence for qRT-PCR and gene marker in this study.

## Data Availability

The gene expression matrix, phenotypic data and the WGCNA results are publicly available at the Open Archive for Miscellaneous Data (https://ngdc.cncb.ac.cn/) under accession number OMIX973. All relevant data are included within the manuscript and its Supporting Information files.

## Abbreviations

ABA: Abscisic acid
ANOVA: Analysis of variance
CS: Chinese Spring
DPA: Days post anthesis
FCGR: First count germination ratio
GI: Weighted germination index
GR: Germination ratio
GS: Gene significance
GWAS: Genome-wide association analysis
ICIM: Inclusive Composite Interval Mapping
KL: Kernel length
KW: Kernel width
MGR: Mean germination rate
PH: Plant height
PHS: Pre-harvest sprouting
PVE: Phenotypic variances
QTLs: Quantitative trait loci
SL: Spike length
TKW: Thousands kernel weight
WGCNA: Weighted gene co-expression network analysis
