# Supplementary material for: Combining QTL mapping and gene co-expression network analysis for prediction of candidate genes and molecular network related to yield in wheat
Source: BMC Plant Biol. 2022 Jun 13;22:288. doi: 10.1186/s12870-022-03677-8 (PMC9190149; doi:10.1186/s12870-022-03677-8)
Supplement: Supplementary file 1 — Additional file 1: Figure S1. Chinese wheat Zhou8425B and Chinese Spring. Figure S2. Frequency distributions of PH, SL, KL, KW and TKW for the RILs in three environments. Figure S3. Correlation analysis among the nine phenotypes. Figure S4. Weighted gene co-expression network analysis for expressed genes of the RILs. Figure S5. Promoter sequence alignment of TaODORANT1 between wheat Chinese spring and Zhou8425B. Figure S6. The full-length agarose gel plots of gene markers. Figure S7. The gene markers on QTL TaSL1 were significantly associated with spike length and kernel length. [file 12870_2022_3677_MOESM1_ESM.pdf]

Figure S1. Chinese wheat Zhou8425B and Chinese Spring.

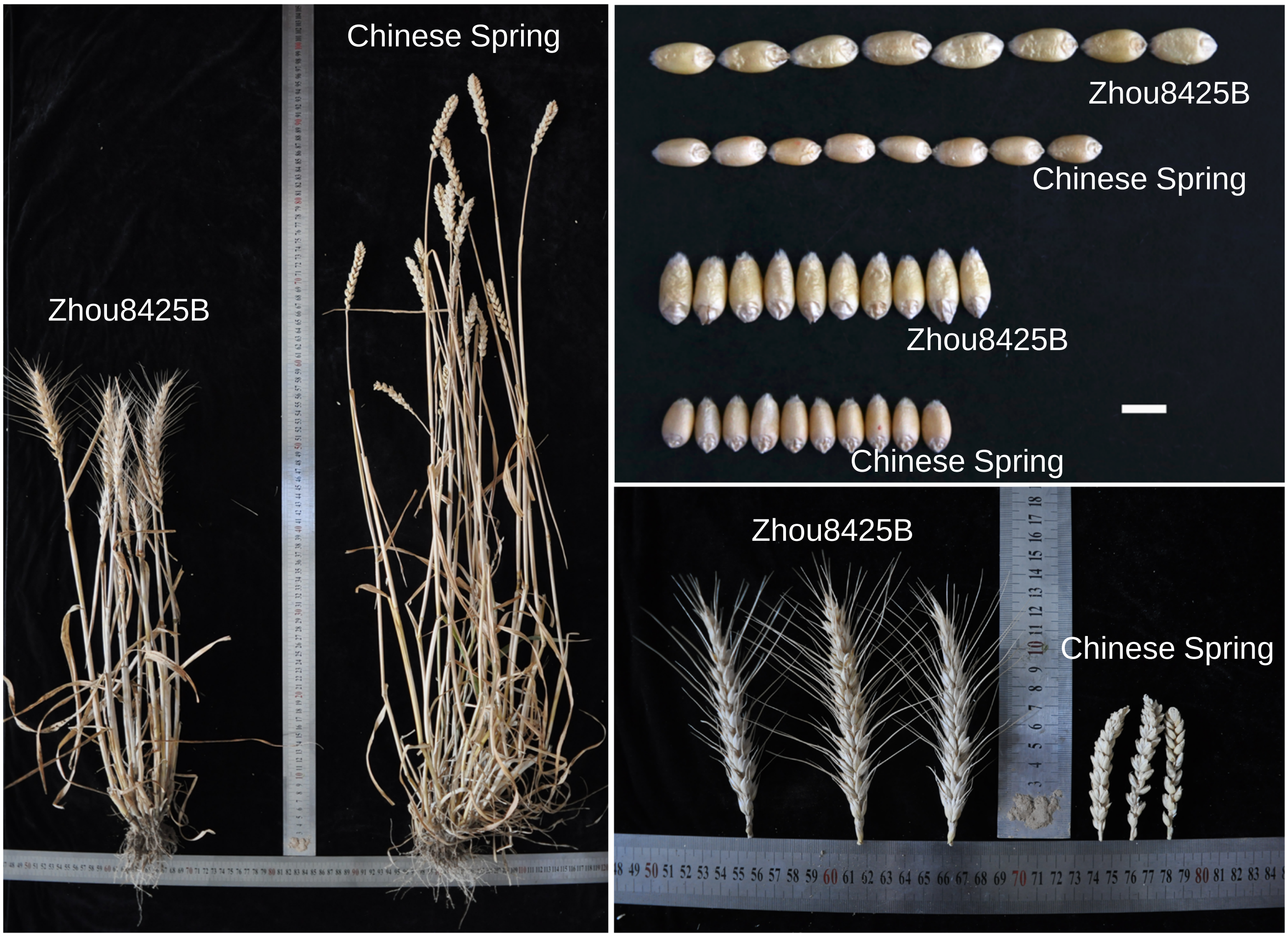

The left photo shows the whole plants with mature spike of Zhou8425B and Chinese Spring. The upper right photo shows the kernels of Zhou8425B and Chinese Spring, and the bar indicates 5mm. The bottoms right photo shows the spikes of Zhou8425B and Chinese Spring.

Figure S2. Frequency distributions of PH, SL, KL, KW and TKW for the RILs in three environments.

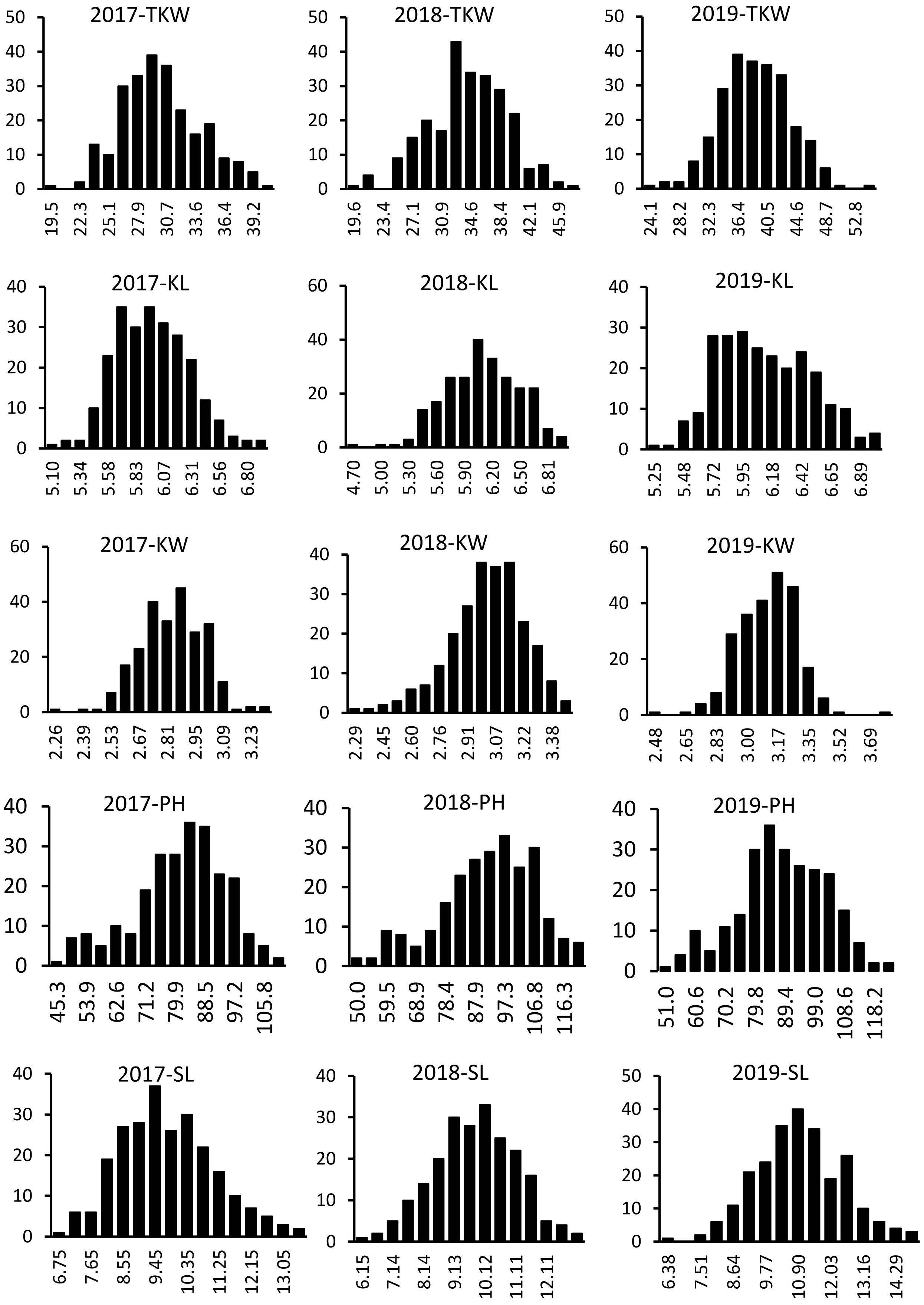

2017, 2018 and 2019 indicates different environments of planting.

Figure S3. Correlation analysis among the nine phenotypes.

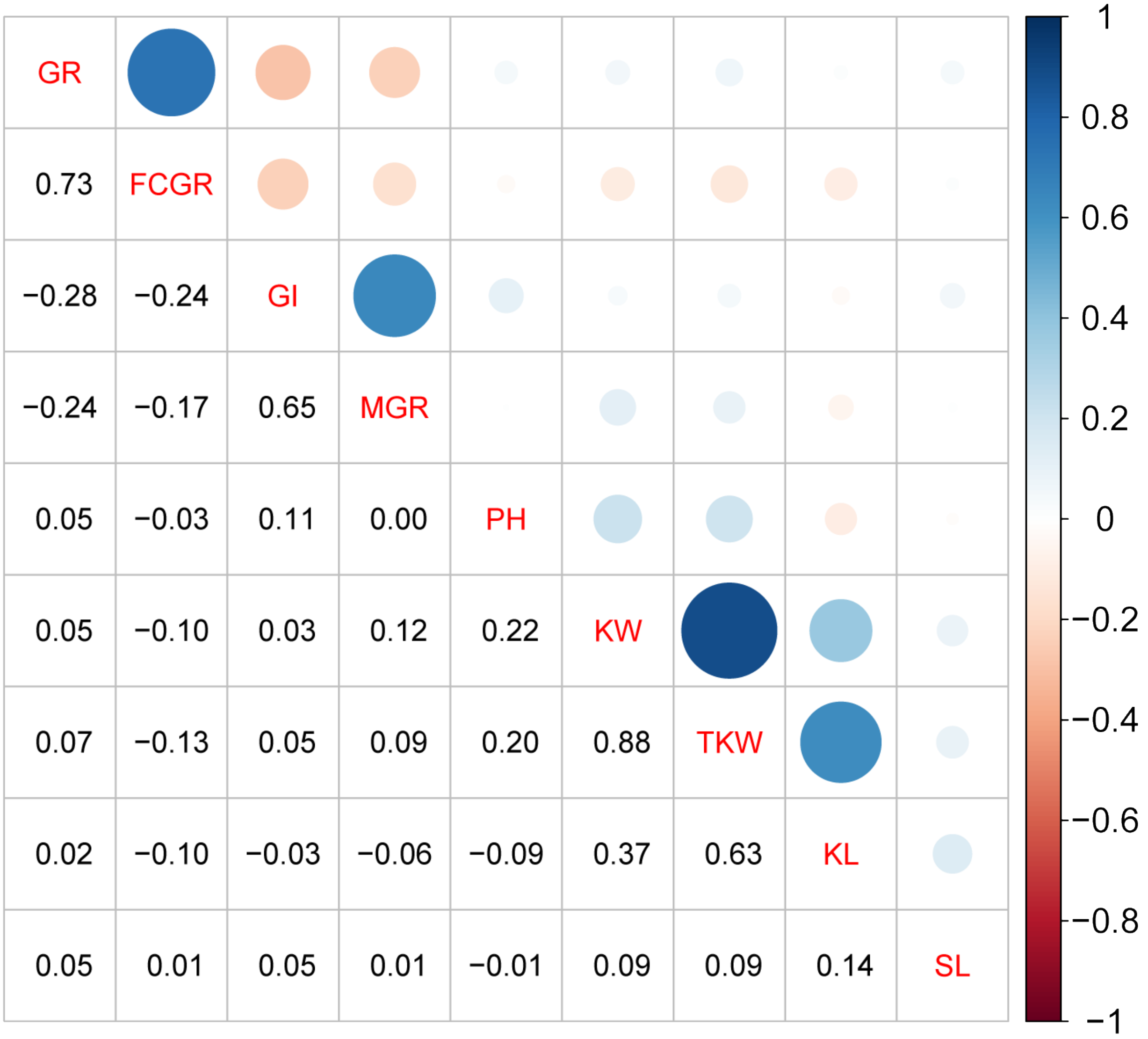

The average value of each phenotype was used for the analysis. The larger the circle graph shows the smaller the *P*-value.

Figure S4. Weighted gene co-expression network analysis for expressed genes of the RILs.

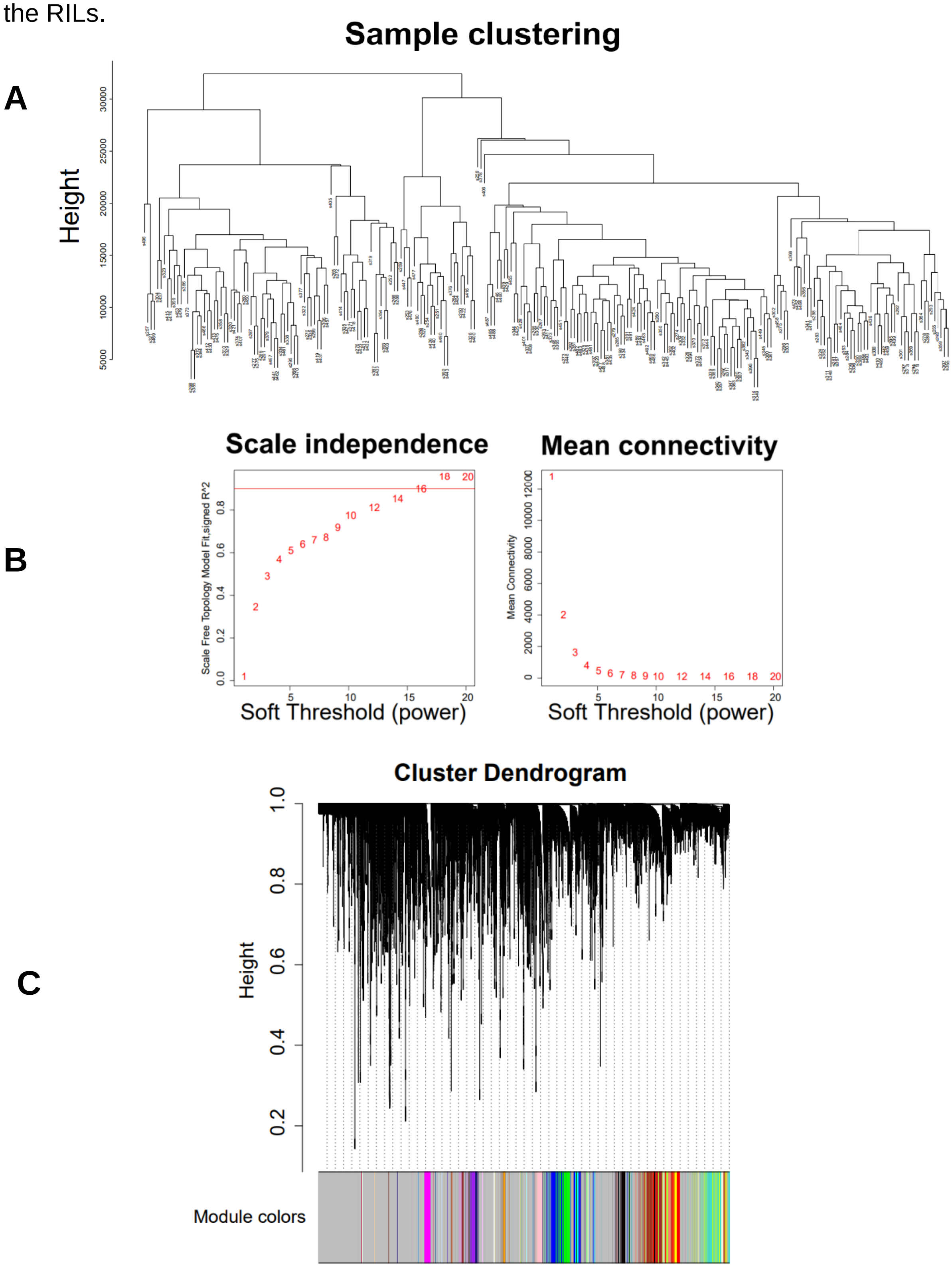

(A) Cluster analysis of the 241 samples based on phenotype using method = "average". (B) Analysis of network topology for various soft-thresholding powers. The left panel shows the scale-free fit index (*y*-axis) as a function of the soft-thresholding power (*x*-axis). The right panel displays the mean connectivity (degree, *y*-axis) as a function of the soft-thresholding power (*x*-axis). (C) Clustering dendrogram of genes, with dissimilarity based on topological overlap, together with assigned module colors.

Figure S5. Promoter sequence alignment of *TaODORANT1* between wheat Chinese spring and Zhou8425B.

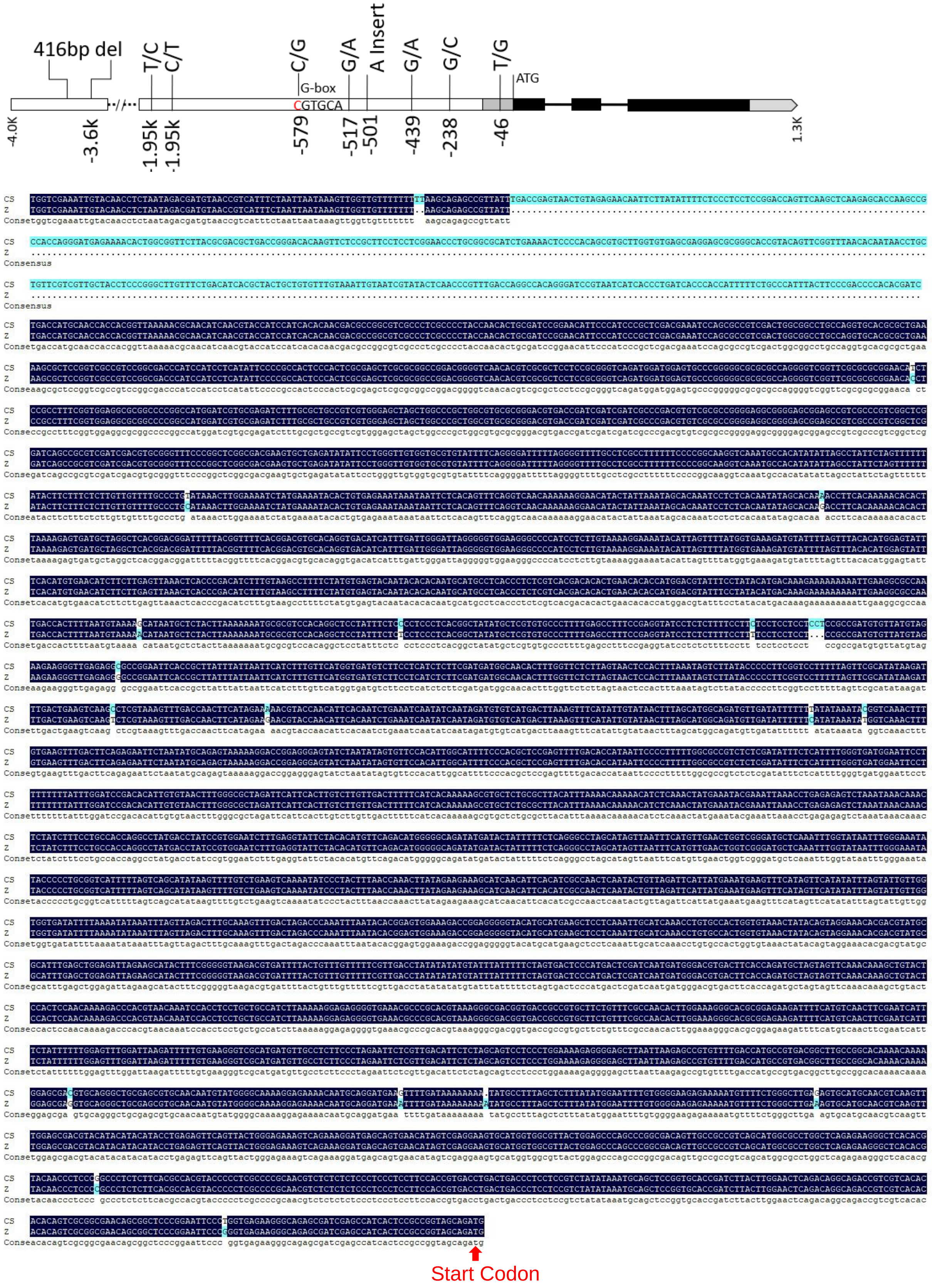

The upper picture solid black box represents exons and the solid black line represents introns; The gray box represents 5'-UTR and the gray arrow box represents 3'-UTR; The white box and dotted line represents promoter.

Figure S6. The full-length agarose gel plots of gene markers.

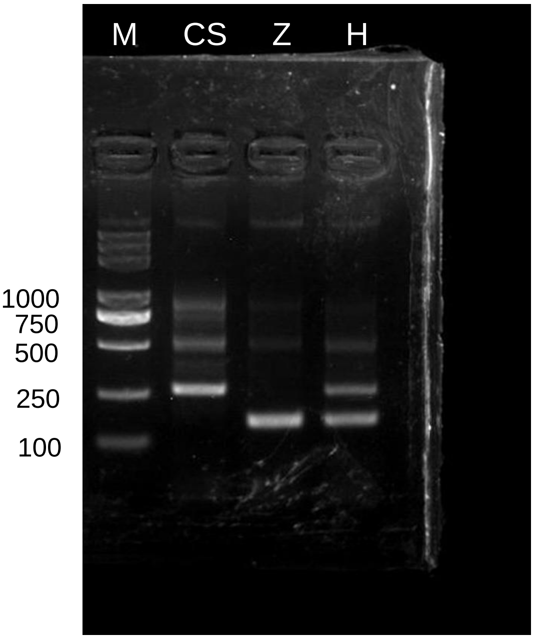

(A) Agarose gel plot of gene marker for *BJ-6840*

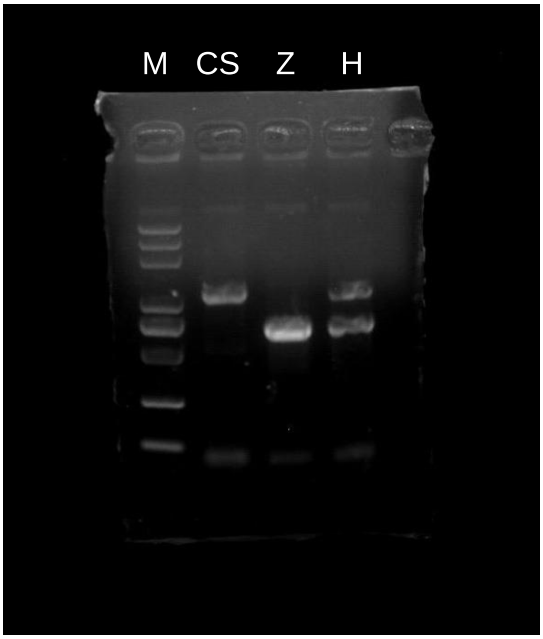

(B) Agarose gel plot of gene marker for *BJ-P2010K*

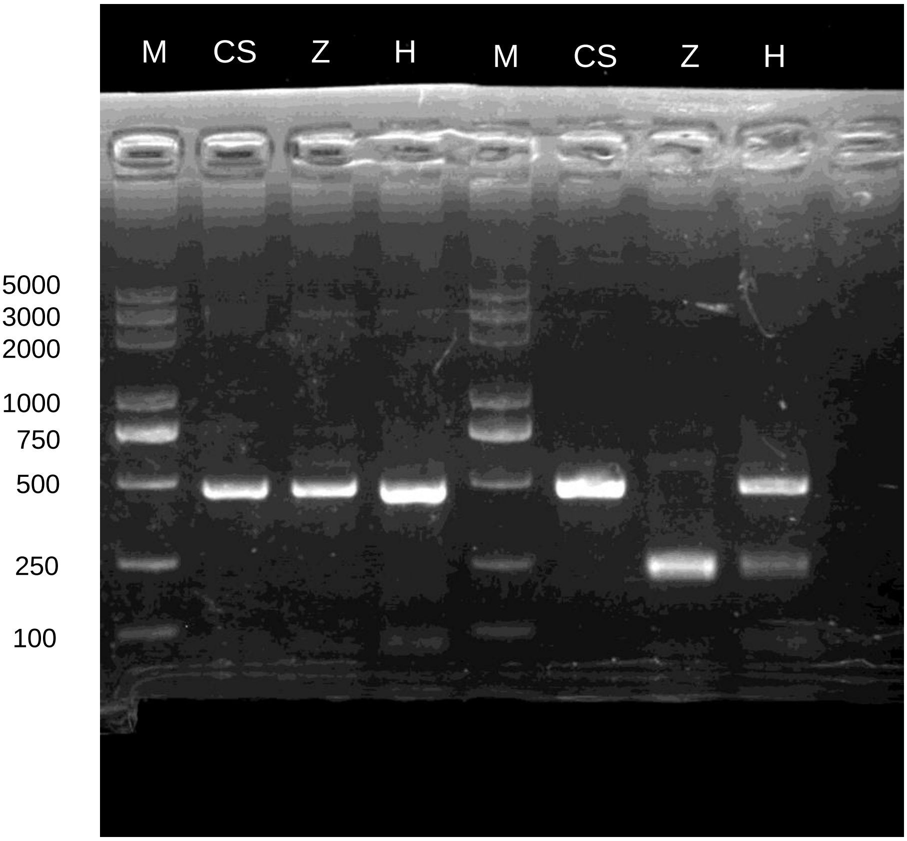

(C) Agarose gel plot of gene marker for *BJ-P2010.* The PCR products of primer set BJ-P2010-F/R are shown on the left . The right are the result of *Sma*I digestion of PCR product

Figure S7. The gene markers on QTL *TaSL1* were significantly associated with spike length and kernel length.

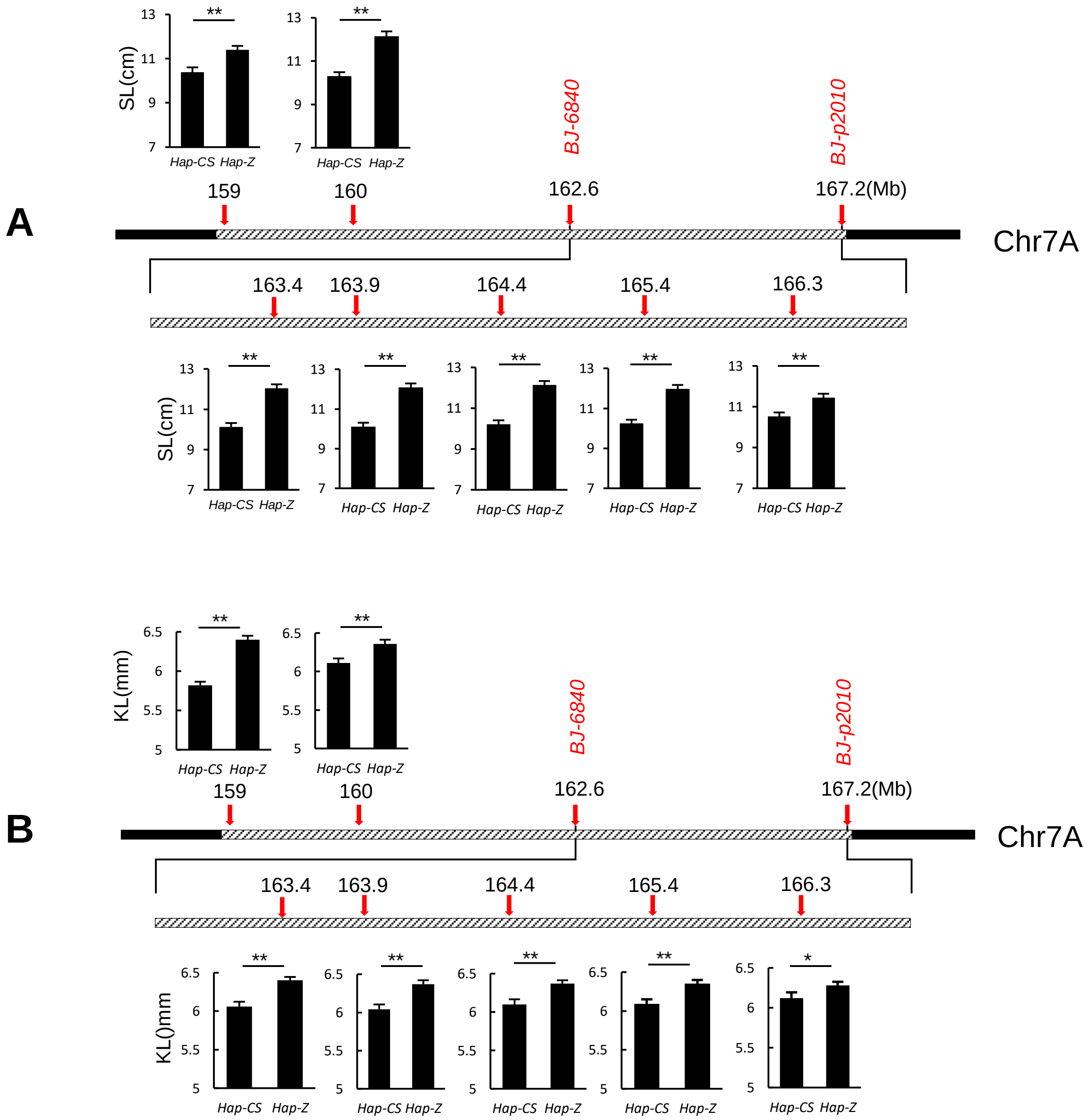

The number indicated by the red arrow is the marker ID, and its physical location as well. (A) Gene markers were associated with spike length. (B) Gene markers were associated with kernel length. Gene marks information is shown in Table S6. Each marker was used for association analysis with 265 wheat landraces. *, two-tailed *t*-test is significant at the 0.05 level. **, two-tailed *t*-test is significant at the 0.01 level. *Hap-CS* and *Hap-Z* represent the two haplotypes derived from Chinese spring and Zhou8425B wheat, respectively.
